# Aiduqing formula inhibits breast cancer metastasis by suppressing TAM/CXCL1-induced Treg differentiation and infiltration

**DOI:** 10.1186/s12964-021-00775-2

**Published:** 2021-08-30

**Authors:** Jing Li, Shengqi Wang, Neng Wang, Yifeng Zheng, Bowen Yang, Xuan Wang, Juping Zhang, Bo Pan, Zhiyu Wang

**Affiliations:** 1 The Research Center of Integrative Cancer Medicine, Discipline of Integrated Chinese and Western Medicine, The Second Clinical College of Guangzhou University of Chinese Medicine, Guangzhou, Guangdong China; 2 Guangdong Provincial Key Laboratory of Clinical Research On Traditional Chinese Medicine Syndrome, Guangdong Provincial Academy of Chinese Medical Sciences, Guangdong Provincial Hospital of Chinese Medicine, Guangzhou, Guangdong China; 3 Guangdong-Hong Kong-Macau Joint Lab On Chinese Medicine and Immune Disease Research, Guangzhou University of Chinese Medicine, Guangzhou, Guangdong China; 4 The Research Center for Integrative Medicine, School of Basic Medical Sciences, Guangzhou University of Chinese Medicine, Guangzhou, Guangdong China; 5 State Key Laboratory of Dampness, Syndrome of Chinese Medicine, The Second Affiliated Hospital of Guangzhou University of Chinese Medicine, Guangzhou, 510120 China

**Keywords:** Aiduqing formula, Tumor-associated macrophage, Chemokine CXCL1, Regulatory T cell, Naive CD4^+^ T cell, Breast cancer metastasis

## Abstract

**Background:**

Metastasis represents the leading cause of death in patients with breast cancer. Traditional Chinese medicine is particularly appreciated for metastatic diseases in Asian countries due to its benefits for survival period prolongation and immune balance modulation. However, the underlying molecular mechanisms remain largely unknown. This study aimed to explore the antimetastatic effect and immunomodulatory function of a clinical formula Aiduqing (ADQ).

**Methods:**

Naive CD4^+^ T cells, regulatory T cells (Tregs), and CD8^+^ T cells were sorted by flow cytometry. Then, breast cancer cells and these immune cells were co-cultured in vitro or co-injected into mice in vivo to simulate their coexistence. Flow cytometry, ELISA, qPCR, double luciferase reporter gene assay, and chromatin immunoprecipitation assay were conducted to investigate the immunomodulatory and antimetastatic mechanisms of ADQ.

**Results:**

ADQ treatment by oral gavage significantly suppressed 4T1-Luc xenograft growth and lung metastasis in the orthotopic breast cancer mouse model, without noticeable hepatotoxicity, nephrotoxicity, or hematotoxicity. Meanwhile, ADQ remodeled the immunosuppressive tumor microenvironment (TME) by increasing the infiltration of tumor-infiltrating lymphocytes (TILs) and cytotoxic CD8^+^ T cells, and decreasing the infiltration of Tregs, naive CD4^+^ T cells, and tumor-associated macrophages (TAMs). Molecular mechanism studies revealed that ADQ remarkably inhibited CXCL1 expression and secretion from TAMs and thus suppressed the chemotaxis and differentiation of naive CD4^+^ T cells into Tregs, leading to the enhanced cytotoxic effects of CD8^+^ T cells. Mechanistically, TAM-derived CXCL1 promoted the differentiation of naive CD4^+^ T cells into Tregs by transcriptionally activating the NF-κB/FOXP3 signaling. Lastly, mouse 4T1-Luc xenograft experiments validated that ADQ formula inhibited breast cancer immune escape and lung metastasis by suppressing the TAM/CXCL1/Treg pathway.

**Conclusions:**

This study not only provides preclinical evidence supporting the application of ADQ in inhibiting breast cancer metastasis but also sheds novel insights into TAM/CXCL1/NF-κB/FOXP3 signaling as a promising therapeutic target for Treg modulation and breast cancer immunotherapy.
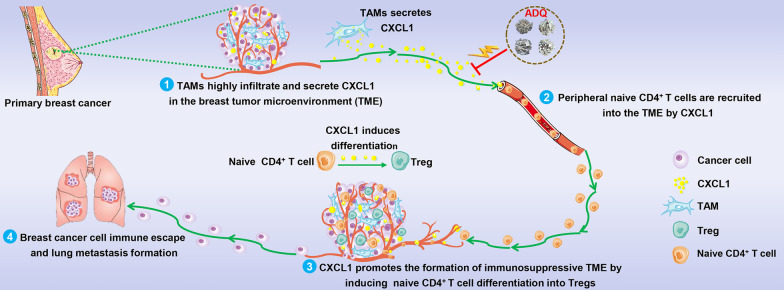

**Video Abstract**

**Supplementary Information:**

The online version contains supplementary material available at 10.1186/s12964-021-00775-2.

## Background

Although the overall survival (OS) and prognosis of breast cancer have remarkably improved in the past decades, breast cancer still represents the most frequently diagnosed malignancy as well as the leading cause of cancer-related deaths among women worldwide [1]. According to the latest global cancer epidemiological data, there were about 2.3 million new cases and 685,000 deaths of breast cancer in 2020 worldwide. Breast cancer alone accounted for 24.5% of all new cancer cases and 15.5% of all cancer deaths among females [1]. Moreover, 90% of breast cancer-related deaths result from distant metastases in the lungs, bone, liver, and brain [2, 3]. Lung metastases are of particular concern as they are associated with 60–70% of breast cancer patients’ mortality [3]. Therefore, it is of great clinical significance and urgent need to further elucidate the biological mechanism driving breast cancer metastasis, and to develop clinically effective antimetastatic drugs.

Notably, most metastasis-related studies in the past decades have focused on genetic alteration-induced cancer cell transformation. In recent years, an increasing number of studies have suggested the immunosuppressive tumor microenvironment (TME) as an important supportive factor in promoting breast cancer metastasis. According to the immune-editing theory, the interaction between tumor cells and the immune system can be divided into three stages: immune elimination, immune equilibrium, and immune escape [4]. Metastasis is a multistep process involving the immune escape of cancer cells from the primary site, survival in the circulation system, dissemination to other organs, and finally colonization at the distant sites [5]. Immune escape of cancer cells from the primary tumor is the first and crucial step for cancer cells to achieve metastasis successfully. Tumor-infiltrating immune cells within the TME play important roles in inducing the immune escape of cancer cells. TME is a dynamic system that consists of multiple kinds of immune cells including helper T cells (e.g., Th1 and Th2), cytotoxic T lymphocytes (CTLs), natural killer cells (NKs), tumor-associated macrophages (TAMs), regulatory T cells (Tregs), and myeloid-derived suppressor cells (MDSCs). In recent years, much more attention has been paid to the targeted modulation of the immune components in the TME [6]. The increased abundance of immunosuppressive components in the TME, including numerous immunosuppressive cells and cytokines, could protect tumor cells from immune surveillance and elimination, eventually leading to immune escape and tumor metastasis. The primary immunosuppressive cell populations infiltrating in the TME include Tregs, TAMs, and MDSCs [7]. Tregs represent a physiologically immunosuppressive subpopulation of T cells and play crucial roles in promoting the establishment of immunosuppressive TME [8]. Peripheral Tregs usually derive from naive CD4^+^ T cells, which differentiate and develop into Tregs under stimulation [9]. Treg infiltration is closely related to the metastasis and poor clinical prognosis of multiple malignant tumors including breast cancer [10, 11]. There is growing evidence that Treg infiltration within the TME could hinder the functions and activities of effector T cells and NK cells by secreting soluble immunosuppressive cytokines (e.g., TGF-β) and expressing inhibitory receptors (e.g., CTLA4), thereby protecting cancer cells from immune elimination. Notably, the existing knowledge about the biological mechanisms underlying Treg differentiation and intratumoral infiltration is still very limited, which remains a major obstacle for the development of Treg-related cancer treatment strategies.

In addition to Tregs, TAMs are also among the most abundant immune cells within the TME. TAMs represent one of the key drivers of immunosuppressive TME formation and cancer metastasis. TAMs can overexpress immunosuppressive molecules (e.g., PD-1/PD-L1) to inhibit the cancer-cell killing function of the effector cells and therefore induce the formation of immunosuppressive TME and tumor metastasis [12]. Additionally, TAMs are highly plastic immune cells capable of activating or polarizing into different phenotypes. M1-like activation causes the release of various cytokines, such as interleukin 6 (IL-6), IL-1β, and TNF-α, all of which facilitate a proinflammatory response [13]. In contrast, M2-like activation could turn on the expression of antiinflammatory cytokines, such as resistin-like molecule α, IL-10, and arginase 1 (ARG1) [14]. TAMs usually exhibit an M2-like phenotype and secrete multiple cytokines and chemokines to modulate cancer cells directly or recruit other types of immunosuppressive cells into the TME [15]. Our previous studies have found that CXCL1 is one of the most abundant chemokines secreted by TAMs [16, 17]. TAM/CXCL1 inhibition represents a promising treatment strategy for preventing breast cancer immune escape and lung metastasis [17, 18]. For example, CXCL1 derived from TAMs could recruit hematopoietic stem and progenitor cells (HSPCs) and induce their differentiation into MDSCs to shape the immunosuppressive TME and favor breast cancer metastasis [19]. Interestingly, there is emerging evidence suggesting that TAMs could also facilitate the intratumoral infiltration of Tregs [20]. However, the underlying molecular mechanism remains to be elucidated. It is important to further investigate whether TAM/CXCL1 signal could also promote breast cancer metastasis by modulating Treg chemotaxis and differentiation.

At present, there are no effective therapeutic drugs for metastatic breast cancer in clinical settings [3]. Chemotherapy remains the first choice for metastatic breast cancer. However, it usually exhibits limited effectiveness and severe side effects in metastatic breast cancer patients [21]. With the encouraging efficacy in improving the survival time [22], reducing the death risk [23], and alleviating side effects [24], traditional Chinese medicine (TCM) has been used empirically for breast cancer treatment and prevention for thousands of years in China and Asian countries. In recent years, TCM has become an essential adjunctive therapy in clinical practice, especially in metastatic or advanced breast cancer patients [25]. ADQ formula was created by Prof. Zhiyu Wang based on the TCM theory and long-term clinical experience [26]. ADQ formula is composed of four herbs including *Oldenlandia diffusa, Curcuma zedoaria, Astragalus membranaceus,* and *Glycyrrhiza uralensis Fisch*. The clinical observation results have shown that ADQ formula could remarkably decrease the risk of metastasis and recurrence in breast cancer patients. Preclinical studies have proven that ADQ extract could chemosensitize breast cancer by directly attenuating caveolin-1 expression in mammary tumor cells [26]. Additionally, ADQ extract could inhibit breast cancer metastasis by inhibiting CXCL1-mediated autophagy [27]. It is worthwhile to further uncover the biological mechanism of ADQ in suppressing breast cancer metastasis to provide preclinical evidence supporting its clinical application. The degradation of extracellular matrix (ECM) by matrix metalloproteinases (MMPs) also represents a critical mechanism for tumor invasion and metastasis [28, 29]. Our previous study has shown that ADQ could inhibit breast cancer metastasis by suppressing epithelial-mesenchymal transition via attenuating MMPs in the TME [27]. Considering that TAM/CXCL1 activity within the TME represents a reliable target for TCM to prevent breast cancer immune escape and metastasis [17, 18], it is important to analyze whether ADQ also suppresses breast cancer metastasis by remodeling the immunosuppressive TME via modulating TAM/CXCL1 activity.

Herein, we systematically demonstrated that ADQ suppressed the immune escape and lung metastasis of breast cancer by remodeling the immunosuppressive TME via suppressing the TAM/CXCL1/Treg pathway. This study reveals the immunomodulatory mechanism of ADQ in inhibiting breast cancer metastasis and highlights the TAM/CXCL1/Treg axis as a promising therapeutic target for breast cancer immunotherapy.

## Materials and methods

### Cell culture and induction

The mouse breast cancer 4T1 cells, mouse macrophages Raw264.7, and human embryonic kidney 293 T cells were purchased from the American Type Culture Collection (ATCC). 4T1-Luc cells were generated by transfecting the lentiviral plasmid harboring the luciferase gene into 4T1 cells. Therefore, 4T1-Luc cells could fluoresce in the presence of D-luciferin substrate. All cell lines have been confirmed by short tandem repeat profiling and were used between passage numbers 10–20. 4T1 cells, 4T1-Luc cells, and 293 T cells were cultured in DMEM complete medium, while Raw264.7 cells, CD8^+^ T cells, Tregs, and naive CD4^+^ T cells were cultured in RPMI-1640 complete medium. Cells were maintained in a humidified incubator containing 5% CO_2_ at 37 °C. 40 ng/ml IL-4 and IL-13 (PeproTech) were used to induce the transformation of Raw264.7 macrophages into M2-like macrophages (Raw264.7-derived TAMs).

### Animal experiments

Female Balb/c mice (18–22 g) were purchased from the Beijing Vital River Laboratory Animal Technology Co., Ltd, and raised in the Experimental Animal Center of Guangdong Provincial Hospital of Chinese Medicine. To generate the 4T1-Luc xenograft model, 2 × 10^6^ 4T1-Luc cells were re-suspended in 200 μl PBS and inoculated subcutaneously into the mammary fat pads of mice. To determine the antimetastatic activity of ADQ in vivo, the tumor-bearing mice were randomized into 3 groups (n = 10), including saline group, 0.7 g/kg/day ADQ group, and 1.4 g/kg/day ADQ group. To investigate whether ADQ suppressed breast cancer metastasis by blocking the TAM/CXCL1 pathway, mice were randomly assigned into 6 groups (n = 10), including saline group (2 × 10^6^ 4T1-Luc cells injection), 0.7 g/kg/day ADQ group (2 × 10^6^ 4T1-Luc cells injection), TAM group (2 × 10^6^ 4T1-Luc cells were co-injected with 6 × 10^6^ Raw264.7-derived TAMs), TAM/shCXCL1 group (2 × 10^6^ 4T1-Luc cells were co-injected with 6 × 10^6^ Raw264.7/shCXCL1-derived TAMs), TAM + 0.7 g/kg/day ADQ group, and the TAM/rCXCL1 + 0.7 g/kg/day ADQ group (2 × 10^6^ 4T1-Luc cells were co-injected with 6 × 10^6^ Raw264.7/rCXCL1-derived TAMs). ADQ was administrated by oral gavage every day. ADQ preparation and quality control analysis were conducted as we previously described [26]. Mice were weighed and tumors were measured every 3 days. Tumor volumes (V) were determined by the formula: V = (length × width^2^)/2. To monitor the growth and lung metastasis of 4T1-Luc xenografts, the IVIS Lumina XR in vivo imaging system (PerkinElmer) was used to photograph the mice. Mice were euthanized by 3% isoflurane when tumors reached a diameter of 2 cm. Then, lungs and tumors were excised and photographed. Primary cells were isolated using a mechanical procedure. Briefly, single cell suspensions were prepared from spleens by slide mechanical grind. The red blood cells in spleen samples were lysed with a Tris-ammonium-chloride-based buffer (BD Biosciences). Then, cells were centrifuged and re-suspended in PBS solution. The re-suspended cells were counted and subjected to flow cytometry to detect or sort the indicated subpopulations including Tregs, naive CD4^+^ T cells, Th1 cells, and TAMs. Lung tissues and the remaining tumor tissues were preserved in 4% paraformaldehyde or a −80 °C refrigerator, and subjected to the tissue immunofluorescence assay or HE staining assay as indicated below. To investigate the hepatotoxicity, nephrotoxicity, or hematotoxicity of ADQ on mice, the blood samples were collected and subjected for biochemical analysis as previously reported [30]. Briefly, mouse serum (1:3 dilution) was obtained by centrifugation and used to analyze the hepatic function parameters (alanine transaminase and aspartate aminotransferase) and renal function parameters (urea, uric acid, and creatinine) using the Automatic Biochemical Analyzer (Roche Group, Basel, Switzerland). The fresh blood samples were collected and used to detect hematological parameters including white blood cell numbers, red blood cell numbers, and hemoglobin content using the Automatic Blood Cell Analyzer (Mindray, Shenzhen, China).

### Flow cytometry assay

The population analysis and sorting of Tregs, naive CD4^+^ T cells, CD8^+^ T cells, and TAMs were performed by flow cytometry using the FACSAria III flow cytometer (BD Biosciences) or NovoCyte flow cytometer (ACEA Biosciences). The antibodies used in flow cytometry assay were as follows: PE-Cy7-conjugated CD45 antibody (25-0451-82, eBioscience), FITC-conjugated F4/80 antibody (SC-71085, Santa Cruz Biotechnology), PE-conjugated CD206 antibody (141706, Biolegend), PE-conjugated CD4 antibody (100407, Biolegend), APC/Cyanine7-conjugated CD8a antibody (100714, Biolegend), PE-Cy7-conjugated CD25 antibody (25-0251-81, Invitrogen), APC-conjugated FOXP3 antibody (17–4777-42, eBioscience), and BV650-conjugated CD45RA antibody (564360, BD Biosciences). The sorted lymphocytes were re-suspended in RPMI-1640 complete culture medium and stimulated by 50 ng/ml PMA, 1 ug/ml ionomycin, and 10 ng/ml IL-2 (Multiscience, Hangzhou, China) in vitro for 24–48 h, and then continuously cultured in RPMI-1640 complete culture medium.

### Immunofluorescence assay

Immunofluorescence assay was performed as previously reported [31]. Briefly, the tissue sections were dewaxed, hydrated, and subjected to antigen retrieval by incubating slides in the boiled citrate buffer (0.01 M, pH 6.0). For cell immunofluorescence assay, cells were cultured in the confocal dish, treated as indicated, and fixed with 4% paraformaldehyde for 20 min. Then, the tissue sections or cells were washed three times with PBS, and permeabilized with 0.25% Triton X-100 for 20 min. After blocking with 5% BSA solution for 1 h, the tissue sections or cells were incubated with primary antibodies overnight, followed by incubation with the secondary antibodies. DAPI was used to visualize the nuclei. Fluorescence images were photographed using the LSM710 confocal microscope (Zeiss, Jena, Germany).

The antibodies used in immunofluorescence assay were as follows: CD206 antibody (141706, Biolegend), CXCL1 antibody (AF5403, Affinity), CD34 antibody (AF5149, Affinity), CD62L antibody (25-0621-82, eBioscience), FOXP3 antibody (17-4777-42, eBioscience), p65 antibody (8242S, CST), CD4 antibody (100407, Biolegend), IFN-γ antibody (BS-0480R, Bioss), RAlexa Fluor® 488 conjugated-anti-rabbit IgG (4412S, CST), Alexa Fluor®555 conjugated-anti-mouse IgG (A21422, Invitrogen), Alexa Fluor® 555 conjugated-anti-rat IgG (4417S, CST), and the Alexa Fluor® 555 conjugated-anti-rabbit IgG (4413S, CST).

### HE staining assay

HE staining assay was applied as previously reported [30] to investigate the effects of ADQ on TILs infiltration and lung metastasis. Briefly, the tissue sections were deparaffinized and hydrated firstly. Then, 10% hematoxylin was used to visualize the cell nucleus, while 1% eosin was used to stain the cytoplasm. Finally, the specimens were dehydrated, cleared, and mounted for microscopic examination. The infiltration levels of TILs in mammary tumors were evaluated according to the 5-step standardized approaches for TILs evaluation [32]. The five steps include (1) selecting the tumor area; (2) defining the stromal area; (3) scanning at low magnification; (4) determining types of inflammatory infiltration; and (5) assessing the percentage of TILs infiltration.

### Cytotoxicity assay

To assess the effects of ADQ on the cell viability of Raw264.7-derived TAMs and naive CD4^+^ T cells, the CCK-8 assay was applied using the CCK8 Kit (C0038, Beyotime Biotechnology). Briefly, cells were seeded into the 96-well plate at a density of 5 × 10^3^ cells/per well. After attachment, cells were treated with serial concentration gradients of ADQ for 12–48 h. After ADQ treatment, 20 μl CCK-8 was added to each well, and cells were incubated for another 2 h. The absorbance of each well was measured at 450 nm using a multifunctional microplate reader.

### ELISA assay

ELISA assay was applied to determine the inhibitory effect of ADQ on CXCL1 secretion from TAMs. TAMs were treated with various concentrations of ADQ (20–200 μg/ml) for 24 h. Then, the cell culture supernatants were collected and subjected to CXCL1 concentration analysis using the Mouse CXCL1 ELISA Kit (SEA041Mu, USCN Business). Briefly, 100 μl cell culture supernatants or standard solutions were added to each well of the CXCL1 antibody-coated 96-well plates. After incubation at 37 °C for 1 h, the plates were washed with cleaning solution for 3 times, followed by incubation with 100 μl detection solution A at 37 °C for 1 h. Then, the plates were rinsed with cleaning solution again, followed by incubation with 100 μl detection solution B at 37 °C for 1 h. Then, the plates were rinsed again, followed by incubation with 90 μl substrate solution at 37 °C for 30 min. Lastly, 100 μl termination solution was added to each well of the plates. The absorbance of the final reactant was measured at 450 nm using a multifunctional microplate reader.

### Western blot

Western blot assay was applied as we previously reported [31]. The following antibodies were used in the Western blot assay including CXCL1 antibody (AF5403, Affinity), β-actin antibody (4970S, CST), p65(10745-I-AP, Proteintech), FOXP3 (22228–1-AP, Proteintech), GAPDH (5174S), IκBα (AF5002, Affinity), p-IκBα (AF2002, Affinity), α-Tubulin (11224–1-AP, Proteintech), Lamin B1 (12987–1-AP, Proteintech), iNOS (18985–1-AP, Proteintech), and ARG1 (DF6657, Affinity).

### qPCR assay

qPCR assay was conducted as we previously reported [31]. The primer sequences of murine *β-ACTIN* gene were 5′-GGAGGGGGTTGAGGTGTT-3′ (forward) and 5′-GTGTGCACTTTTATTGGTCTCAA-3′ (reverse). Primer sequences of murine *CXCL1* gene were 5′-GACTCCAGCCACACTCCAAC-3′ (forward) and 5′-TGACAGCGCAGCTCATTG-3′ (reverse). Primer sequences of murine *FOXP3* gene were 5′-CACCTATGCCACCCTTATCCG-3′ (reverse) and 5′-CATGCGAGTAAACCAATGGTAGA-3′ (reverse).

### Double luciferase reporter gene assay

Double luciferase reporter gene assay was performed as we previously reported [18] to investigate the promoter activities of *CXCL1* and *FOXP3* genes under indicated stimulations. Briefly, the *CXCL1* and *FOXP3* promoter plasmids (Genecopeia) were respectively transfected into Raw264.7-derived TAMs and naive CD4^+^ T cells using Vigenefection (Vigene Biosciences). After treatment as indicated, *CXCL1* and *FOXP3* promoter activities were detected using the Secrete-Pair™ Dual Luminescence Assay Kit (LF031, Genecopeia).

### CFSE staining assay

CFSE staining assay was performed to determine the proliferation of CD8^+^ T cells. Briefly, CD8^+^ T cells were seeded in the 96 well plates, and stained with CSFE (C1031, Beyotime) for 15 min. Subsequently, cells were centrifuged and re-suspended with RPMI-1640 medium. After treatment as indicated, CD8^+^ T cells were collected and analyzed by flow cytometry.

### Chemotaxis assay

Chemotaxis assay was conducted using the 24-well transwell chambers with polycarbonate filters (5 μm pore size). Briefly, the bottom chamber was firstly filled with the complete culture medium. Then, 5 × 10^4^ naive CD4^+^ T cells were isolated from murine spleens, re-suspended with serum-free culture medium, and seeded in the upper chamber. Exogenous stimulations were administered to the bottom chamber. After treatment as indicated, CD4^+^ T cells in the bottom chamber were enumerated by flow cytometry.

### Co-culture of Tregs, CD8^+^ T cells and 4T1 cells

Tregs, CD8^+^ T cells as well as 4T1 cells were obtained as described above and co-cultured in RPMI-1640 complete culture medium at a ratio of 1:10:2. After treatment as indicated, the non-adherent T cells were harvested. The proliferation and apoptosis of the CD8^+^ T cell population were detected by CFSE-PI staining and flow cytometry. The expression levels of perforin and granzyme B in CD8^+^ T cells were analyzed by flow cytometry using the PE-conjugated perforin antibody (12-9392-82, eBioscience) and APC-conjugated granzyme B antibody (17-8898-82, eBioscience). The adherent 4T1 cells were harvested and subjected to LDH activity detection using the LDH Activity Kit (A020-2-2, Jiancheng Bioengineering Institute).

#### Chromatin immunoprecipitation (CHIP) -PCR assay

To determine whether CXCL1 could induce the binding of NF-κB subunit p65 to the promoter region of *FOXP3*, CHIP assay was conducted by immune-precipitating the DNA fragments with p65 antibody (8242S, CST) using the CHIP Assay Kit (P2078, Beyotime) under the manufacturer’s instructions [33]. The + 151 to + 160 promoter region of the *FOXP3* gene (NC_000086.8:7445915-7461482) was predicted as the binding site of p65 by the hTFtarget database. This region in the immune-precipitated DNA samples was amplified by PCR assay using the primers of 5′-ATCCCCCTCTAGCAGTCCAC-3′ (forward) and 5′-GGACCAACTGCTCCACCTAT-3′ (reverse).

### TUNEL assay

TUNEL assay was conducted as we previously described [30] using the TUNEL Kit (C1088, Beyotime). Briefly, paraffin-embedded tumor sections were deparaffinized and treated with proteinase K to strip proteins from the nuclei. Then, the tissue sections were saturated in 3% hydrogen peroxide for 10 min, incubated with terminal deoxynucleotidyl transferase at 37 °C for 1 h, followed by incubation with streptavidin-FITC at 37 °C for 30 min. Lastly, the apoptosis of tumor tissue was detected using the confocal microscope (LMS710, ZEISS).

### Statistical analysis

Statistics were calculated using the SPSS 26.0 software. Data were expressed as mean ± standard deviation. One-way analysis of variance (ANOVA) and Student’s t-test were used for pairwise comparisons. *p* < 0.05 was considered statistically significant.

## Results

### ADQ inhibits lung metastasis and remodels the immunosuppressive TME of breast cancer

**Fig. 1:**
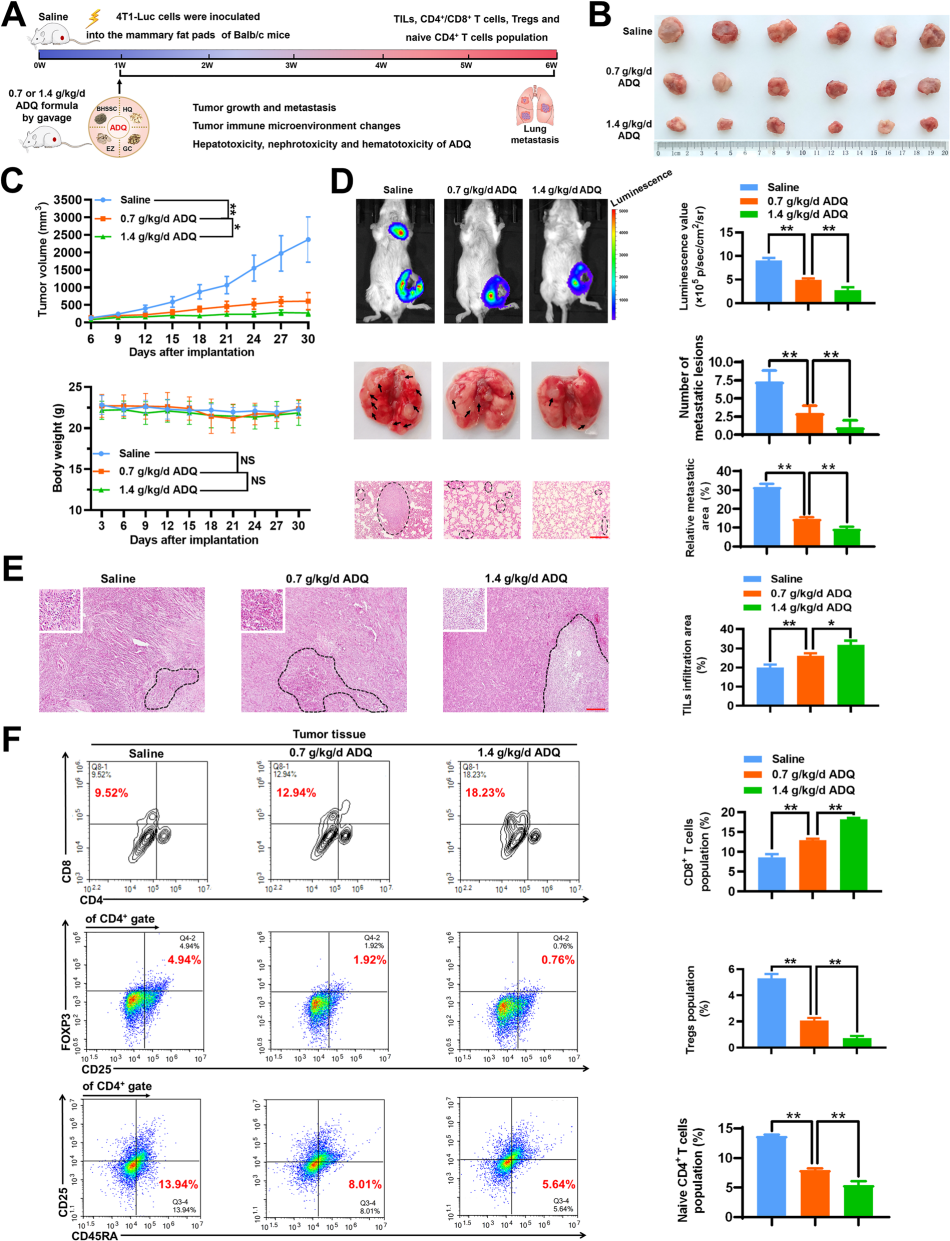
ADQ inhibits lung metastasis and the immunosuppressive TME of breast cancer. **a** The schematic diagram of the animal assay. 4T1-Luc metastasis-tracking model of breast cancer was established by inoculating 2 × 10^6^ 4T1-Luc cells into the mammary fat pads of Balb/c mice. Saline or ADQ (0.7 or 1.4 g/kg/day) was administrated by oral gavage. **b** The representative pictures of tumors in each group. N = 6. **c** Tumor volume (top) and mouse weight (bottom) curves. N = 6. **d** The lung metastasis of breast cancer xenograft was detected by the in vivo imaging assay and lung HE staining assay. The arrows and circles indicate the metastatic tumor foci in murine lungs. Scale bar = 100 μm. N = 3. **e** The representative HE staining pictures of tumor tissues. The infiltration levels of tumor-infiltrating lymphocytes (TILs) in breast tumors of each group were evaluated following the 5-step standardized approaches. Scale bar = 100 μm. N = 3. **f** The infiltration levels of cytotoxic CD8^+^ T cells, CD4^+^/CD25^+^/FOXP3^+^ Tregs, and CD4^+^/CD25^−^/CD45RA^+^ naive CD4^+^ T cells within the TME were quantified by flow cytometry. N = 3. *p < 0.05; **p < 0.01

Our previous study has shown that ADQ formula or paclitaxel treatment alone could moderately suppress the growth of breast tumors in the MMTV-PyVT^±^ breast cancer spontaneous tumor-forming mice, while their combination achieves a synergistic inhibition effect on that [26]. Breast cancer 4T1 cells are a highly metastatic cell line with a tendency to form lung metastasis in vivo. To investigate the antimetastatic and immunomodulatory effects of ADQ in vivo, the 4T1-Luc metastasis-tracking model of orthotropic breast cancer was established by planting the luciferase-expressing 4T1-Luc cells into the mammary fat pads of Balb/c mouse. According to the equivalent dosage conversion between humans and mice, ADQ was administrated at the dosages of 0.7 g/kg/day or 1.4 g/kg/day by oral gavage in the present study (Fig. 1a). ADQ was extracted and quality-controlled as we had previously reported [26]. ADQ administration at dosages of 0.7 g/kg/day or 1.4 g/kg/day significantly delayed the growth of 4T1-Luc xenografts, while no ADQ treatment-related mortality or significant reduction in body weights were detected (Fig. 1b, c). Meanwhile, ADQ treatment did not lead to significant effects on the hepatic and renal function parameters or blood parameters of mice (Additional file 1: Table 1), suggesting that ADQ exhibited no noticeable hepatotoxicity, nephrotoxicity, or hematotoxicity in vivo. Furthermore, the in vivo imaging assay and HE staining assay clearly showed that ADQ remarkably inhibited the lung metastasis of breast cancer, as reflected by the reductions in the areas and numbers of lung metastatic lesions (Fig. 1d). Immune modulation is the central mechanism of TCM in inhibiting tumor growth and progression. HE staining assay showed that ADQ significantly elevated the infiltration level of TILs within the TME (Fig. 1e). The composition of TILs within the TME determines whether its phenotype favors immune evasion or antitumor immunity [34]. Therefore, to investigate the composition changes of TILs after ADQ treatment, immune cell population analysis of the tumor tissue was performed. Cytotoxic CD8^+^ T cells are the key effector cells to strengthen the anti-cancer immune response within the TME, while Tregs are the key immune cell subpopulation in shaping the immunosuppressive TME. ADQ treatment significantly increased the infiltration of cytotoxic CD8^+^ T cells whereas decreasing the infiltration of immunosuppressive CD4^+^/CD25^+^/FOXP3^+^ Tregs within the TME (Fig. 1f). Naive CD4^+^ T cells have been reported to differentiate into Tregs under exogenous stimuli [9]. ADQ also significantly suppressed the infiltration of CD4^+^/CD25^−^/CD45RA^+^ naive CD4^+^ T cells (Fig. 1f), whereas increasing the infiltration of CD4^+^/IFN-γ^+^ Th1 cells within the TME (Additional file 1: Fig. 1). Altogether, ADQ not only inhibits the lung metastasis of breast cancer in vivo but also remodels the immunosuppressive TME.

### ADQ inhibits M2 phenotype polarization and CXCL1 secretion by TAMs in vitro and in vivo

**Fig. 2:**
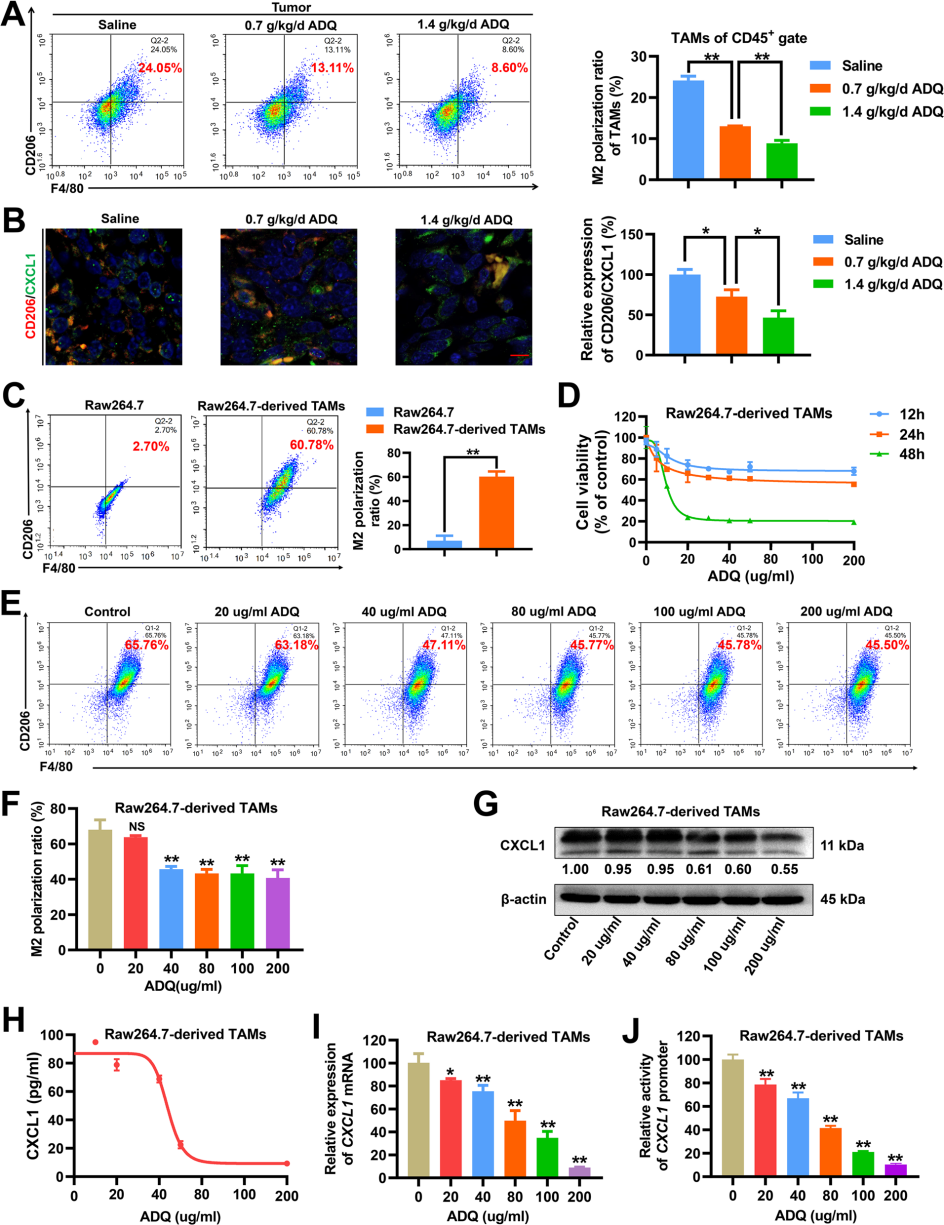
ADQ inhibits M2 phenotype polarization and CXCL1 secretion by TAMs in vitro and in vivo*.* **a** Flow cytometry assay was conducted to investigate the effect of ADQ treatment (0.7 or 1.4 g/kg/day) on TAM infiltration and polarization within the TME of breast tumors. **b** The expression levels of CD206 (red) and CXCL1 (green) in breast tumor tissues were detected by the tissue immunofluorescence assay. Scale bar = 5 μm. **c** 40 ng/ml IL-4 and IL-13 were used to induce the transformation of Raw264.7 macrophages into M2-like macrophages (TAMs). Their phenotype validation was conducted by flow cytometry. **d** The cell viability changes of TAMs when treated with ADQ (20–200 μg/ml) for 12–48 h were detected using the CCK-8 assay. **e**–**f** The phenotype changes of Raw264.7-derived TAMs when treated with 20–200 μg/ml ADQ for 24 h were detected by flow cytometry. **G**–**i** Raw264.7-derived TAMs were treated with ADQ (20–200 μg/ml) for 24 h. The protein expression, secretion as well as mRNA transcription levels of CXCL1 in Raw264.7-derived TAMs were detected by Western blot, ELISA, and qPCR assays, respectively. **j** Raw264.7-derived TAMs were treated with ADQ (20–200 μg/Ml) for 24 h. The promoter activity changes of *CXCL1* gene in Raw264.7-derived TAMs were detected by the double luciferase reporter gene assay. N = 3. *p < 0.05. ^**^p < 0.01

TAMs remain one of the most abundant immune cell subpopulations in breast cancer [35], and CXCL1 is among the most abundant chemokines secreted by TAMs [16, 36]. Our previous studies have indicated that inhibiting the TAM/CXCL1 activity within the TME significantly suppressed breast cancer immune escape and metastasis [17, 19]. Thus, we further investigated the modulatory effect of ADQ on TAM/CXCL1 activity. TAMs usually exhibit an M2 phenotype, which promotes tumor growth and metastasis. As shown in Fig. 2a, ADQ treatment (0.7 or 1.4 g/kg/day) significantly decreased the infiltration and the M2 phenotype polarization of TAMs within the TME. Immunofluorescence assay indicated that ADQ significantly decreased the CD206 (M2 phenotype marker) and CXCL1 expression levels in breast tumor tissues, suggesting that ADQ was able to significantly inhibit TAM infiltration and attenuate CXCL1 expression in vivo (Fig. 2b). Additionally, ADQ also elevated the infiltration of M1-like macrophages within the TME (Additional file 1: Fig. 2). To further validate the inhibitory effect of ADQ on the TAM/CXCL1 pathway, Raw264.7 macrophages were transformed into M2-like macrophages (Raw264.7-derived TAMs) by recombinant murine IL-4 and IL-13 induction (Fig. 2c) and then subjected to ADQ treatment. TAM proliferation was significantly suppressed when treated with 20–200 μg/ml ADQ for 48 h, but not significant at 12 or 24 h (Fig. 2d). Furthermore, ADQ treatment for 24 h remarkably inhibited the M2 phenotype polarization of Raw264.7-derived TAMs in a concentration-dependent manner (Fig. 2e, f). Western blot, ELISA and qPCR assays further confirmed that ADQ suppressed CXCL1 protein expression, secretion as well as mRNA transcription in TAMs (Fig. 2g–i). Finally, the double luciferase reporter gene assay revealed that ADQ inhibited *CXCL1* promoter activity in TAMs (Fig. 2j). In summary, ADQ induces the polarization transformation of the M2-like macrophages into the M1-like phenotype, accompanied by downregulation of CXCL1 expression and secretion.

### TAM/CXCL1 signaling promotes the differentiation and the immunosuppressive function of Tregs

**Fig. 3:**
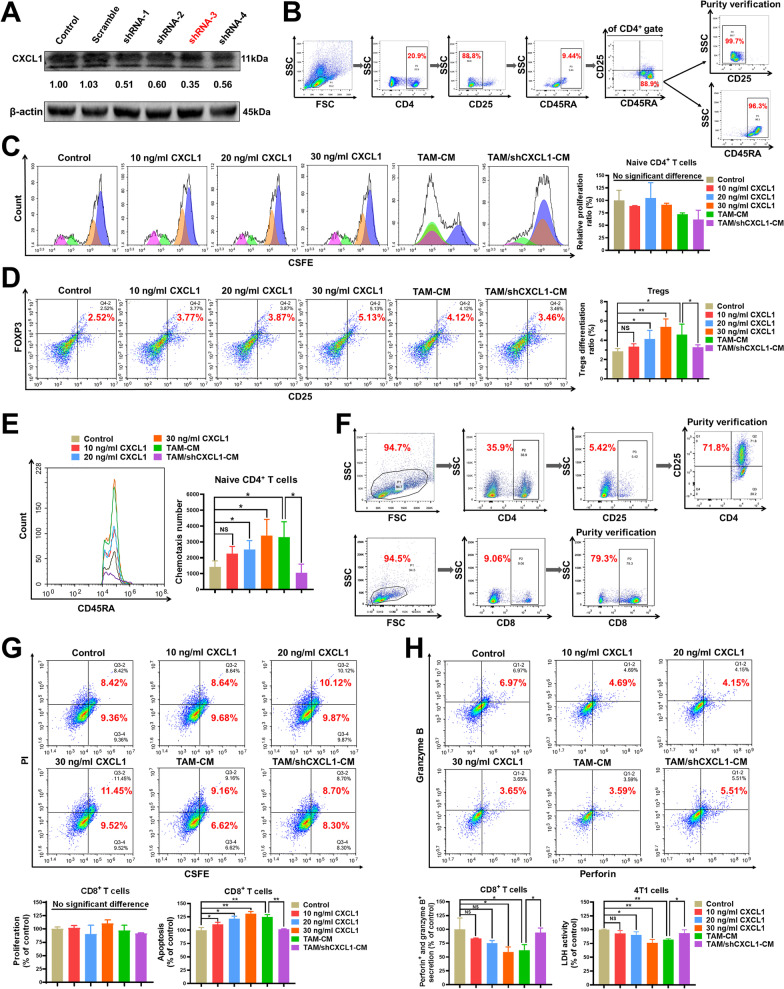
TAM/CXCL1 signaling promotes the differentiation and the immunosuppressive function of Tregs. **a** TAM/shCXCL1 cells were generated by transfecting Raw264.7-derived TAMs with CXCL1 shRNAs. **b** Naive CD4^+^ T cells (CD4^+^/CD25^−^/CD45RA^+^) were sorted from murine spleens by fluorescence-activated cell sorting technology. **c** The proliferation of naive CD4^+^ T cells when treated as indicated for 24 h was investigated by CFSE staining assay and flow cytometry. **d** The differentiation levels of naive CD4^+^ T cells into Tregs when treated as indicated for 24 h were detected by flow cytometry. **e** Chemotaxis assay was conducted to investigate the chemotaxis efficacy of naive CD4^+^ T cells. **f** The sorting strategies of Tregs and CD8^+^ T cells. Tregs and CD8^+^ T cells were sorted from murine spleens by fluorescence-activated cell sorting technology. **g–h** Tregs, cytotoxic CD8^+^T cells, and 4T1 cells were co-cultured at a ratio of 1: 10: 2 and treated as indicated for 24 h. The proliferation activities, apoptosis (**g**), as well as granzyme B and perforin secretion levels **h** of CD8^+^T cells were detected by flow cytometry. The LDH activity of the 4T1 cells (**h)** was investigated using the colorimetric assay. N = 3. *p < 0.05; **p < 0.01

As stated above, ADQ inhibited TAM/CXCL1 activity and decreased the infiltration of Tregs and naive CD4^+^ T cells to remodel the immunosuppressive TME. It has been reported that TAMs could facilitate the intratumoral infiltration of Tregs [20]. However, the underlying mechanism is still unknown. Previous studies have revealed that TAMs can secrete chemokines to recruit other types of immunosuppressive cells into the TME [15]. Therefore, we hypothesized that the repressed TAM/CXCL1 activity after ADQ treatment would further decrease Treg infiltration by suppressing the naive CD4^+^ T cell chemotaxis and differentiation into Tregs. To test this hypothesis, the TAM/shCXCL1 cells were generated by knocking down CXCL1 expression in Raw264.7-derived TAMs (Fig. 3a). Meanwhile, naive CD4^+^ T cells were sorted from murine spleens by fluorescence-activated cell sorting technology (Fig. 3b). As shown in Fig. 3c, TAM/CXCL1 signaling exhibited no effects on naive CD4^+^ T cell proliferation. However, similar to the effect of recombinant murine CXCL1, the conditioned medium (CM) of TAMs was able to significantly induce the differentiation of naive CD4^+^ T cells into Tregs, while CXCL1 knockdown in TAMs (TAM/shCXCL1-CM) partially abrogated that effect (Fig. 3d). Chemotaxis assay indicated that TAM-CM promoted the chemotaxis of naive CD4^+^ T cells, while CXCL1 knockdown in TAMs partially abrogated that effect (Fig. 3e). These results supported our hypothesis that inhibiting TAM/CXCL1 signaling may decrease Treg infiltration by suppressing the naive CD4^+^ T cell chemotaxis and differentiation into Tregs. In addition to Treg development, the effect of TAM/CXCL1 signaling on the immunosuppressive function of Tregs was also investigated. To achieve this, first, Tregs and effector CD8^+^ T cells were sorted from murine spleens (Fig. 3f). Then, Tregs were treated with recombinant murine CXCL1, TAM-CM, or TAM/shCXCL1-CM for 24 h. Subsequently, Tregs were collected and co-cultured with cytotoxic CD8^+^T cells and 4T1 cells for 24 h at a ratio of 1: 10: 2. Finally, the CD8^+^ T cells and 4T1 cells were sorted and subjected to activity analysis. Tregs exert immunosuppressive activity by inducing apoptosis or function inactivation of cytotoxic CD8^+^ T cells. Perforin and granzyme B are the most important cytolytic effector molecules in CD8^+^ T cells against infection and cancer cells [37]. LDH activity is a common biomarker that reflects cell injury. As shown in Fig. 3g, h, pretreatment of Tregs with TAM/CXCL1 signal did not affect the proliferation activities of the co-cultured CD8^+^T cells, but it significantly induced their apoptosis and cytolytic function inactivation, leading to the decreased cytotoxic effect of CD8^+^ T cells on cancer cells. Altogether, TAM/CXCL1 signaling can promote the differentiation and the immunosuppressive function of Tregs in vitro.

### CXCL1 recruits peripheral naive CD4^+^ T cells and induces their differentiation into Tregs by activating the NF-κB/FOXP3 pathway

**Fig. 4:**
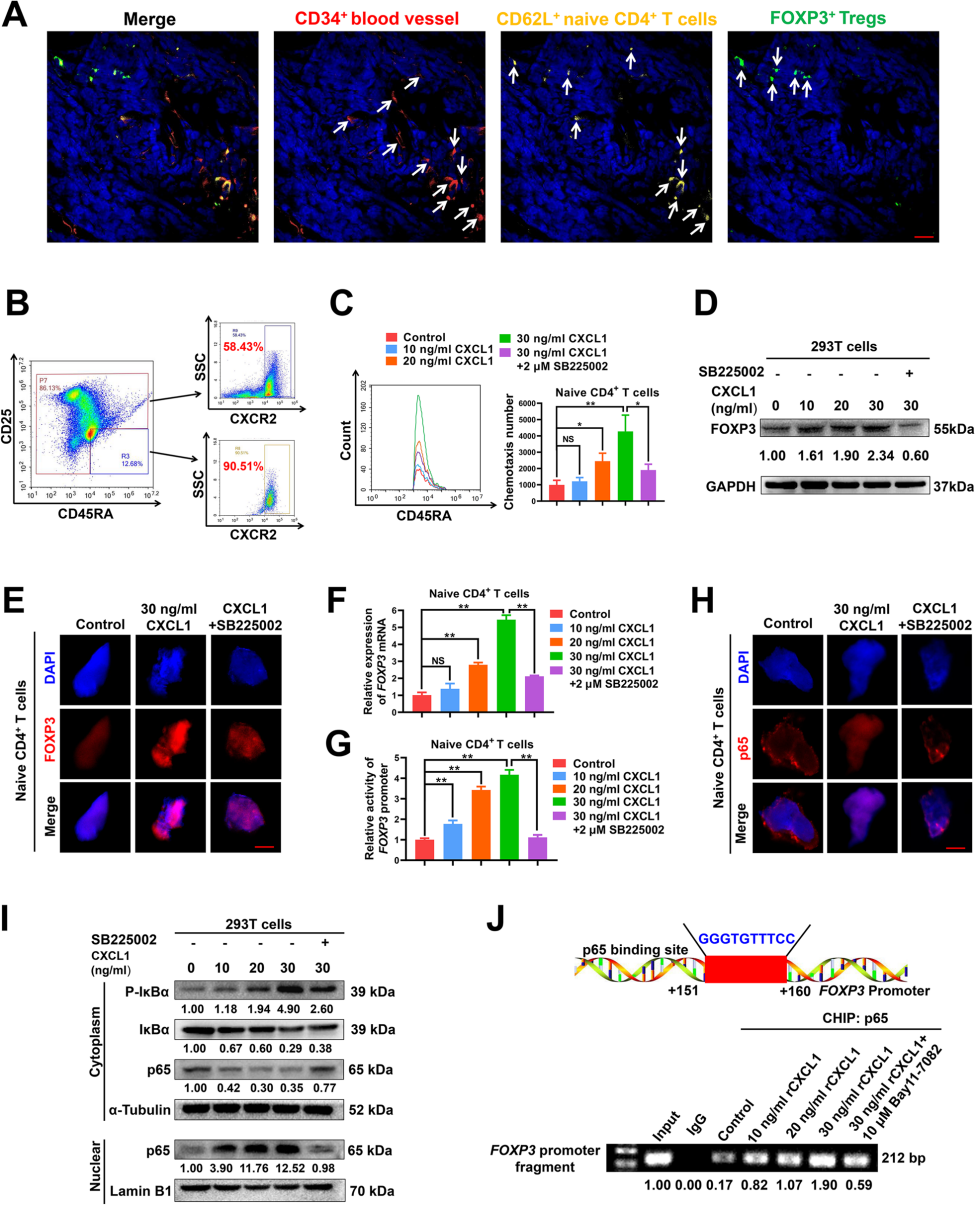
CXCL1 recruits peripheral naive CD4^+^ T cells and induces their differentiation into Tregs in situ by activating the NF-κB/FOXP3 pathway. **a** The distributions of CD34^+^ blood vessels (red), CD62L^+^ naive T cells (yellow), and FOXP3^+^ Tregs (green) within the TME of mammary tumors were detected using the tissue immunofluorescence assay. Scale bar = 5 μm. **b** The expression levels of CXCR2 in naive CD4^+^ T cells sorted from the mammary tumors were detected by flow cytometry. **c** Flow cytometry assay indicated that 10–30 ng/ml CXCL1 treatment for 24 h significantly increased the chemotaxis efficacy of naive CD4^+^ T cells, whereas CXCL1/CXCR2 blockage by 2 μM SB225002 (CXCR2 inhibitor) partially abrogated that effect. **d–e** The expression levels of FOXP3 in 293 T cells (**d**) and naive CD4^+^ T cells (**e**) when treated as indicated for 24 h were detected by Western blot assay and immunofluorescence assay, respectively. Scale bar = 3 μm. **f–g** The mRNA expression level (**f**) and the promoter activity of *FOXP3* gene in naive CD4^+^ T cells when treated as indicated for 24 h were detected by qPCR and double luciferase reporter gene assay, respectively. **h–i** Western blot assay (**h**) and cell immunofluorescence assay (**i**) indicated that CXCL1 treatment (10–30 ng/ml) for 24 h induced the expression and nuclear translocation of NF-κb subunit p65, while CXCL1/CXCR2 blockage partially abrogated that effect. Scale bar = 3 μm. **j** CHIP-PCR assay validated that CXCL1 treatment (10–30 ng/ml) for 24 h promoted the binding of NF-κb subunit p65 with *FOXP3* promoter region, while 10 μM Bay11-7082 treatment (NF-κb inhibitor) partially abrogated that effect. N = 3. *p < 0.05; **p < 0.01

Next, we investigated the molecular mechanisms underlying the promotion effects of CXCL1 on naive CD4^+^ T cell chemotaxis and differentiation into Tregs. Immunofluorescence assay indicated that the CD62L^+^ naive T cells were mainly located around blood vessels within the mammary tumor tissue, whereas Tregs were distributed relatively far away from blood vessels (Fig. 4a). Meanwhile, more than 90% of naive CD4^+^ T cells sorted from the mammary tumors exhibited CXCR2 (CXCL1 receptor) expression, which was relatively higher than that in non-naive CD4^+^ T cells (Fig. 4b). The gradient concentrations of CXCL1 (10–30 ng/ml) significantly increased the chemotaxis efficacy of naive CD4^+^ T cells, while CXCL1/CXCR2 blockage by 2 μM SB225002 (CXCR2 inhibitor) partially abrogated that effect (Fig. 4c). These results indicated that CXCL1 may recruit the peripheral CXCR2^+^ naive CD4^+^ T cells into the breast tumor. FOXP3 is the key modulator molecule that determines Treg differentiation and function. Therefore, we focused on the modulatory effect of CXCL1 on FOXP3 expression to elucidate the underlying mechanism of CXCL1 in inducing naive CD4^+^ T cell differentiation into Tregs. Because of the quantity limitation of the sorted naive CD4^+^ T cells, human embryonic kidney 293 T cells were used in Western blot assay for the analysis of FOXP3 expression modulation. It was found that CXCL1 treatment significantly induced the protein (Fig. 4d, e) and mRNA expression (Fig. 4f) levels of FOXP3 by activating the promoter activity of *FOXP3* gene (Fig. 4g). Subsequently, the modulatory mechanism by which CXCL1 activated the promoter activity of *FOXP3* was investigated in depth. As predicted by the hTFtarget database, there was a putative NF-κb-binding site (5'-GGGGCTTTCC-3', 151 to 160 bp) [38, 39] downstream to the transcription start site of *FOXP3* promoter region. Further studies indicated that CXCL1 was able to induce p65 (NF-κb subunit) expression and nuclear translocation (Fig. 4h, i), while CXCL1/CXCR2 blockage could partially abrogate that effect. CHIP-PCR assay validated that CXCL1 promoted the binding of NF-κb subunit p65 with *FOXP3* promoter region, while 10 μM Bay11-7082 treatment (NF-κb inhibitor) partially abrogated that effect (Fig. 4j). These results indicate that CXCL1 can induce p65 expression and nuclear translocation, thereby transcriptionally elevating *FOXP3* expression and promoting Treg differentiation. Altogether, CXCL1 can recruit the peripheral CXCR2^+^ naive CD4^+^ T cells and induce their differentiation into Tregs in situ by activating the NF-κB/FOXP3 pathway.

### ADQ inhibits TAM/CXCL1-induced differentiation and immunosuppressive function of Tregs in vitro

**Fig. 5:**
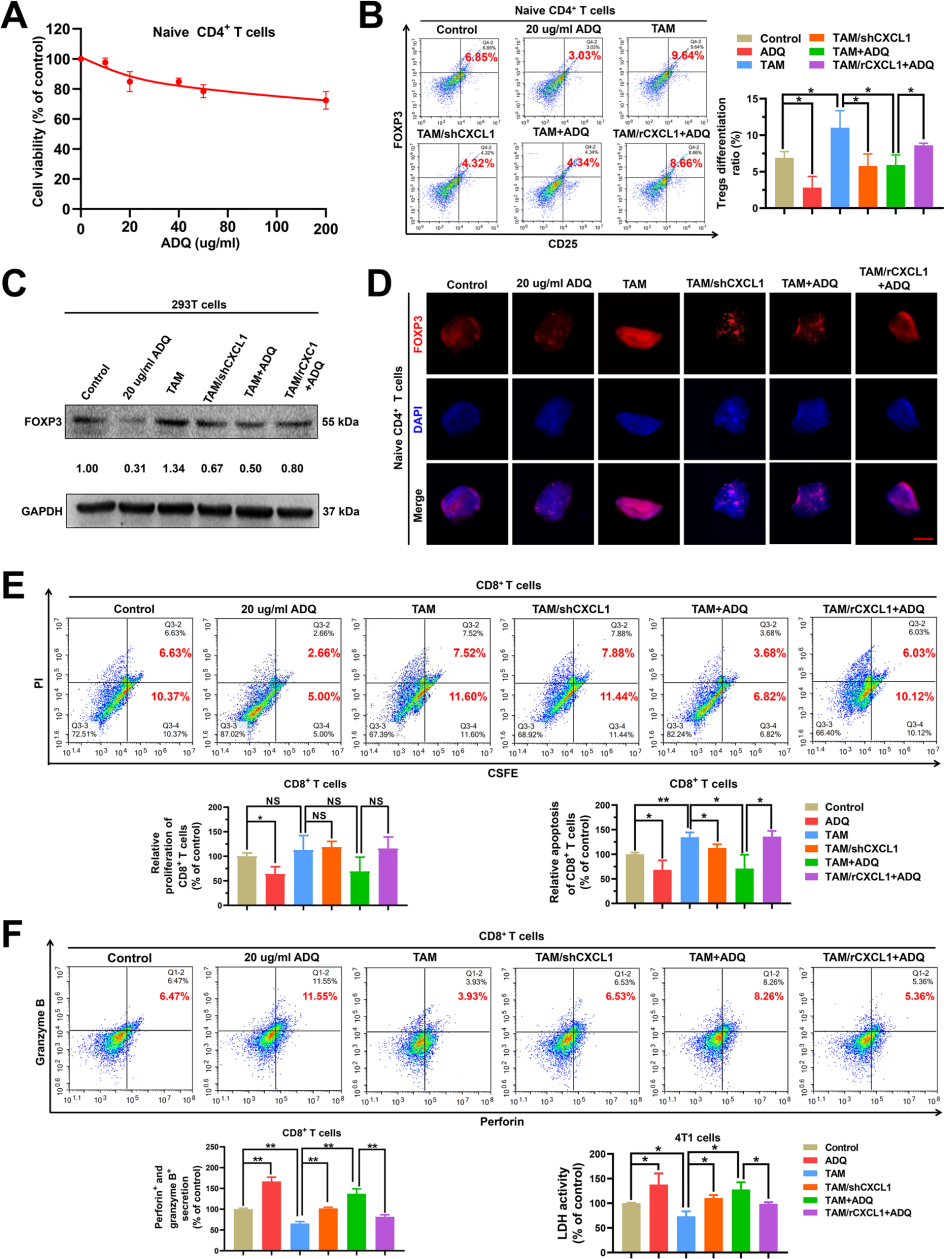
ADQ inhibits TAM/CXCL1-induced differentiation and immunosuppressive function of Tregs in vitro*.* **a** CCK-8 assay indicated that 20–200 μg/ml ADQ treatment for 24 h exhibited a moderate inhibitory effect on naive CD4^+^ T cell proliferation. **b** The differentiation changes of naive CD4^+^ T cells when treated as indicated for 24 h were detected by flow cytometry. **C–d** 293 T cells (**c**) and naive CD4^+^ T cells (**d**) were treated as indicated for 24 h. Western blot (**c**) and immunofluorescence assays (**d**) were conducted to detect the FOXP3 expression levels in 293 T cells and naive CD4^+^ T cells. Scale bar = 3 μm. **e–f** Flow cytometry assay indicated that pretreatment of TAMs with 20 μg/ml ADQ for 24 h partially abrogated TAM/CXCL1-induced immunosuppressive function of Tregs on CD8^+^ T cells. The proliferation activities, apoptosis (**e**), as well as granzyme B and perforin secretion levels (**f**) of CD8^+^T cells were detected by flow cytometry. The LDH activity of the 4T1 cells (**f**) was investigated by the colorimetric assay. N = 3. *p < 0.05; **p < 0.01

Next, we investigated whether suppressing TAM/CXCL1-induced Treg differentiation and infiltration is the central mechanism of ADQ in inhibiting breast cancer immune escape and metastasis. To prove this, we first investigated the effect of ADQ on TAM/CXCL1-induced differentiation and immunosuppressive function of Tregs in vitro. As shown in Fig. 5a, ADQ at concentrations ranging from 20 to 200 ug/ml only exhibited a moderate inhibitory effect on naive CD4^+^ T cell proliferation. However, ADQ significantly inhibited TAM/CXCL1-induced differentiation of naive CD4^+^ T cells into Tregs (Fig. 5b). The mechanistic studies indicated that ADQ significantly inhibited TAM/CXCL1-induced FOXP3 expression in both the 293 T cells (Fig. 5c) and naive CD4^+^ T cells (Fig. 5d). To investigate the modulatory effect of ADQ on TAM/CXCL1-induced immunosuppressive function of Tregs, TAMs or TAM/rCXCL1 cells were pretreated with ADQ for 24 h. Then, their CM was collected and used for Treg treatment for 24 h. Subsequently, Tregs were collected and added to the co-culture system of 4T1 cells and cytotoxic CD8^+^ T cells. It was found that pretreatment of TAMs with ADQ partially reversed TAM/CXCL1-induced immunosuppressive function of Tregs on CD8^+^ T cells, leading to the decreased apoptosis and increased cytotoxic function of CD8^+^ T cells (Fig. 5e–f). Altogether, ADQ can inhibit TAM/CXCL1-induced differentiation and immunosuppressive function of Tregs in vitro.

### ADQ inhibits breast cancer immune escape and lung metastasis by suppressing the TAM/CXCL1/Treg pathway

**Fig. 6:**
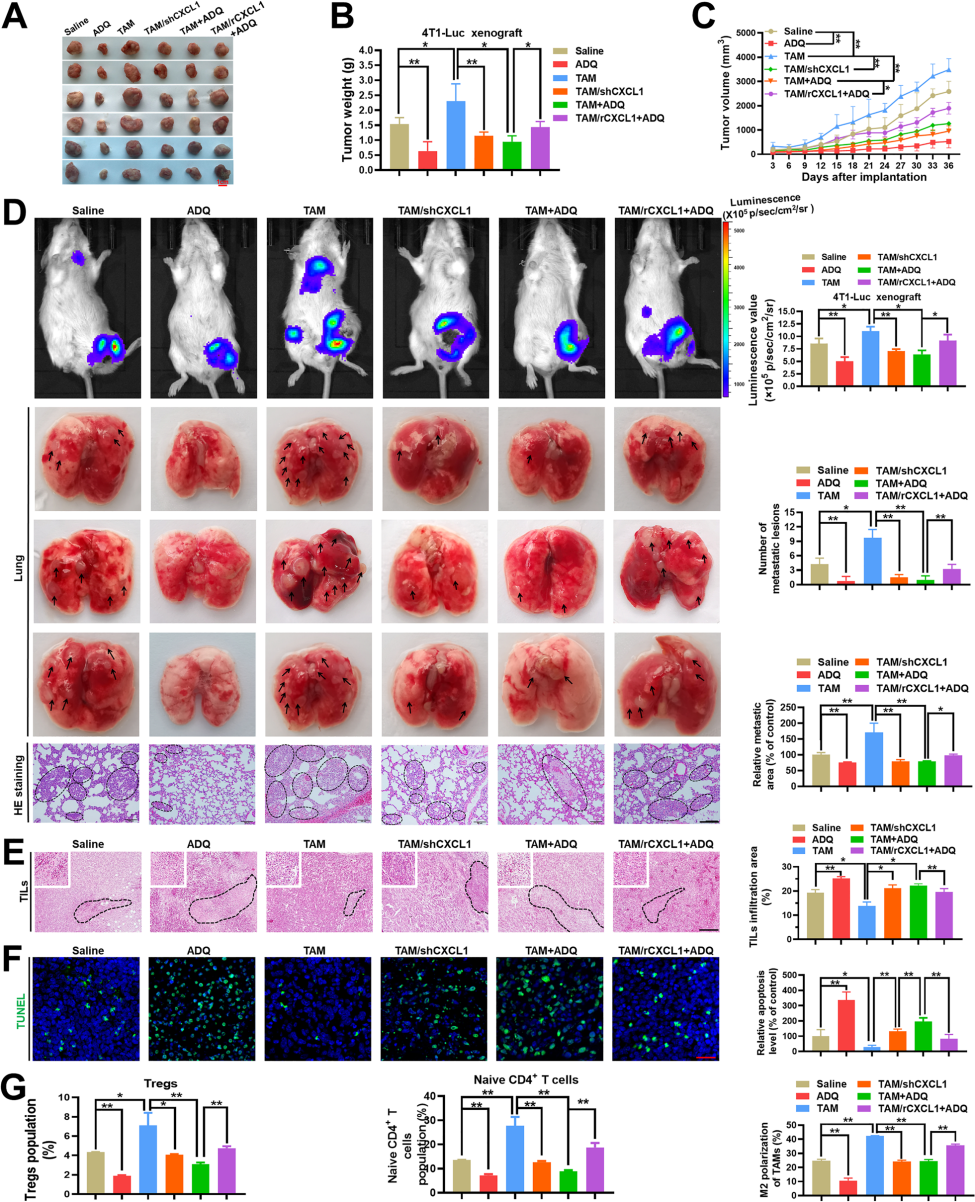
ADQ inhibits breast cancer immune escape and lung metastasis in vivo by suppressing the TAM/CXCL1/Treg pathway. **a–c **ADQ inhibits mammary tumor growth by suppressing TAM/CXCL1 activity. TAM/rCXCL1 refers to the overexpression of CXCL1 in the co-injected TAMs, while TAM/shCXCL1 refers to the knockdown of CXCL1 in the co-injected TAMs. **a** The representative pictures of tumors in each group. N = 6. **b** Tumor weights of each group. N = 10. **c** Tumor volume curves of each group. N = 10. **d** Both the in vivo imaging assay and lung HE staining assay suggested that ADQ dramatically suppressed the lung metastasis of mammary tumors by inhibiting the TAM/CXCL1 pathway. Scale bar = 100 μm. N = 3. Arrows and circles indicate the metastatic tumor foci in murine lungs. **e–f** TIL infiltration level (**e**) and tissue apoptosis (**f**) in mammary tumors were detected by HE staining assay and immunofluorescence assay, respectively. Scale bars indicate 100 μm in € and 20 μm in (**f**). N = 3. **g** The infiltration levels of Tregs, naive CD4^+^ T cells, and TAMs in mammary tumors of each group were detected by flow cytometry. N = 3. *p < 0.05; **p < 0.01

Finally, in vivo studies were conducted to investigate whether ADQ inhibited breast cancer immune escape and lung metastasis by suppressing the TAM/CXCL1/Treg pathway. ADQ administration (0.7 g/kg/day) significantly delayed the growth of mammary tumors, while TAM co-injection remarkably accelerated the growth. Meanwhile, CXCL1 knockdown in the co-injected TAMs partially attenuated the promotion effect of TAMs on breast cancer growth, while CXCL1 overexpression in the co-injected TAMs was demonstrated to accelerate breast cancer growth and metastasis in the zebrafish xenotransplantation model. More importantly, ADQ treatment also significantly delayed TAM co-injection-induced mammary tumor growth, while CXCL1 overexpression in the co-injected TAMs partially abrogated that effect (Fig. 6a–c, Additional file 1: Fig. 3). These results indicate that ADQ can delay breast cancer growth by attenuating TAM/CXCL1 activity. Similarly, both the in vivo imaging assay and HE staining assay suggested that ADQ significantly suppressed breast cancer lung metastasis, which was abrogated by CXCL1 overexpression in co-injected TAMs (Fig. 6d). In terms of the immunosuppressive TME, ADQ significantly elevated the infiltration of TILs within the TME (Fig. 6e) and induced cancer cell apoptosis (Fig. 6f) by inhibiting TAM/CXCL1 activity. Furthermore, ADQ administration significantly reduced the infiltration levels of Tregs and naive CD4^+^ T cells, which were closely correlated with TAM/CXCL1 activity (Fig. 6g). In summary, ADQ can suppress breast cancer immune escape and lung metastasis by blocking the TAM/CXCL1/Treg pathway.

## Discussion

Breast cancer has surpassed lung cancer as the most commonly diagnosed malignancy and the leading cause of cancer deaths among women worldwide [1]. More than 90% of breast cancer-related deaths result from metastatic disease. Metastatic breast cancer is almost incurable, and the 5-year survival rate of metastatic breast cancer patients is only 23.4% [40]. Therefore, great efforts have been made to develop novel treatment options for metastatic breast cancer. Despite extensive efforts, the treatment drugs for metastatic breast cancer are still very limited in the clinic. Currently, chemotherapy is still the first-line treatment option for metastatic breast cancer. However, chemotherapy usually has limited effectiveness in metastatic breast cancer patients because of chemoresistance [21], and its toxicity and side effects (e.g., myelosuppression, gastrointestinal side effects) are severe. In recent years, advances in cancer immunotherapy, such as immune checkpoint inhibition or adoptive cell therapy, have revolutionized the treatment landscapes for multiple metastatic cancers (e.g., metastatic melanoma) [41]. However, breast cancer is considered immunologically cold and immunotherapy usually achieves poor response in breast cancer patients. The results of the vast majority of breast cancer immunotherapy trials were disappointing because of the poor infiltration by lymphocytes and the low mutation burden within the breast cancer TME [42]. Therefore, immunotherapy is still in its infancy for breast cancer. To date, the FDA has only approved the atezolizumab and abraxane combination treatment for PD-L1-positive, unresectable, locally advanced, or metastatic triple-negative breast cancer [43, 44]. With proven clinical efficacy and few adverse effects, TCM has long been used empirically to treat and prevent breast cancer. In particular, TCM is used as an essential adjunctive therapy for metastatic or advanced breast cancer patients in China and neighboring countries [25]. For example, it has been reported that more than 35.6% of breast cancer patients in Taiwan used to undergo TCM treatment [25]. A cohort study has revealed that TCM treatment could dramatically reduce the risk of breast cancer-associated mortality [23]. Regulation of the immune microenvironment has been commonly accepted as the core mechanism by which TCM inhibits tumor growth and metastasis. Herein, we demonstrated that ADQ exhibited no detectable side effects in vivo, while it remarkably suppressed breast cancer immune escape and lung metastasis by suppressing the TAM/CXCL1/Treg pathway. These findings not only provide scientific evidence for the clinical use of ADQ in the prevention and treatment of breast cancer metastasis but also highlight the potential advantage of TCM in remodeling immunosuppressive TME and inhibiting cancer metastasis. Nevertheless, more preclinical studies and clinical trials are warranted to further verify the clinical efficacy of ADQ in the future.

In this study, by using the *in vitr*o co-culture system and the in vivo co-injection methods, TAM/CXCL1 within the TME was identified as the pivotal target of ADQ in inhibiting breast cancer immune escape and lung metastasis. TAMs usually occupy about 30%–50% of the total tumor stromal cells and are among the most abundant immune cell types in numerous tumors including breast cancer [35]. TAMs usually secrete chemokines as inflammatory factors, which recruit more immunosuppressive cells to shape the immunosuppressive TME and favor metastasis [35]. CXCL1 is among the most abundant chemokines secreted from TAMs, and its level in mammary tumor tissue is significantly elevated compared with that in normal breast tissue [16]. A number of studies have revealed that CXCL1 expression elevation in breast stroma usually predicts poor OS and recurrence-free survival (RFS) of breast cancer patients [16, 45]. CXCL1 derived from TAMs can induce breast cancer growth and metastasis through various mechanisms, such as by inducing epithelial-mesenchymal transformation [16], inducing the self-renewal of cancer stem cells (CSCs) [17, 18], promoting autophagy [46], inducing MDSCs infiltration, as well as inducing the formation of the premetastatic niche (PMN) [19]. Targeting TAMs represents a promising antitumor strategy. In recent years, TAM-targeted therapeutics have been rapidly explored and developed. Some examples include blockade of monocytes/macrophage recruitment, pharmacological depletion of TAMs, reprogramming TAMs into antitumor M1-like macrophages, and neutralizing TAM-secreted inflammatory chemokines [15, 47]. In this study, ADQ was shown to exhibit numerous modulatory effects on TAMs, such as inhibiting TAM infiltration, reshaping TAM polarization, attenuating CXCL1 expression, and secretion from TAMs, as well as inhibiting TAM/CXCL1-mediated Treg infiltration. These results strongly suggest TAM/CXCL1 as the target of ADQ in suppressing breast cancer metastasis. Nevertheless, the material basis underlying the targeting modulation effect of ADQ on TAM/CXCL1 has been poorly understood and requires further in-depth investigations. Additionally, considering the complexity of the immune cells and chemokines within the TME, more insights into the modulatory effects of ADQ on other kinds of immune cells (e.g., Th1 cells) or chemokines are still necessary.

The successful development of therapeutic strategies for tumor metastasis is highly dependent on the discovery of the molecular mechanisms of tumor immune escape. Cancer cells can evade immune killing or elimination through various mechanisms, such as reducing immunogenicity (e.g., selective antigen loss), releasing immunosuppressive cytokines, inactivating T cells by overexpressing immune checkpoint molecules, and releasing tumor antigen molecules to block the antibody-dependent cell-mediated cytotoxicity (ADCC) effect [48]. Recently, emerging evidence has emphasized the pivotal role of immunosuppressive TME in favoring the immune escape of tumor cells [49]. As the natural immunosuppressive subpopulation within the TME, Tregs can negatively regulate the intensity and duration of antitumor immune responses by inducing the function inactivation or apoptosis of NK cells and cytotoxic CD8^+^T cells. Therefore, Tregs are key mediators for shaping the immunosuppressive TME [8]. Treg infiltration is closely associated with the invasion, metastasis, and poor clinical prognosis of multiple malignancies, including breast cancer [10, 11]. Tregs could arise either early in ontogeny in the thymus (natural Tregs) or by induction in the periphery (induced Tregs) [50]. Peripheral Tregs usually derive from naive CD4^+^ T cells, which differentiate into Tregs under exogenous stimuli [9]. Inhibition of Treg differentiation and intratumoral chemotaxis are promising strategies to inhibit the formation of immunosuppressive TME. However, to date, the existing knowledge about the modulatory mechanism of Treg differentiation and intratumoral infiltration has been very limited, which represents an obstacle for the successful development of Treg-related cancer treatment strategies. For example, Deng et al*.* reported that TAMs can facilitate Treg infiltration in mammary tumors [20]. However, the underlying mechanisms remained unknown. Herein, we firstly demonstrated that CXCL1 derived from TAMs recruited CXCR2^+^ naive CD4^+^ T cells into the mammary TME and induced their differentiation into Tregs to establish the immunosuppressive TME. This finding is consistent with Song’s report that Tregs in mammary tumors mainly derive from naive CD4^+^ T cells [51]. FOXP3 is the key modulator molecule of Treg differentiation and function. In terms of molecular mechanisms, CXCL1 induced the expression and nuclear translocation of NF-κb subunit p65 in naive CD4^+^ T cells, which bound to the 5'-GGGTGTTTCC-3' region of *FOXP3* promoter and transcriptionally elevated *FOXP3* expression. This finding is consistent with the existing reports that 5'-GGGTGTTTCC-3' region is the putative NF-κb-binding site [38, 39]. These results highlight TAM/CXCL1 as a druggable target for inhibiting Treg activity within the TME. Therefore, CXCL1 neutralizing antibody or CXCR2 receptor inhibitors may represent the promising candidate drugs for Treg-mediated tumor immune escape and metastasis, which have received increasing research attention in recent years and are under preclinical or clinical trials [15, 52, 53].

## Conclusion

**Fig. 7:**
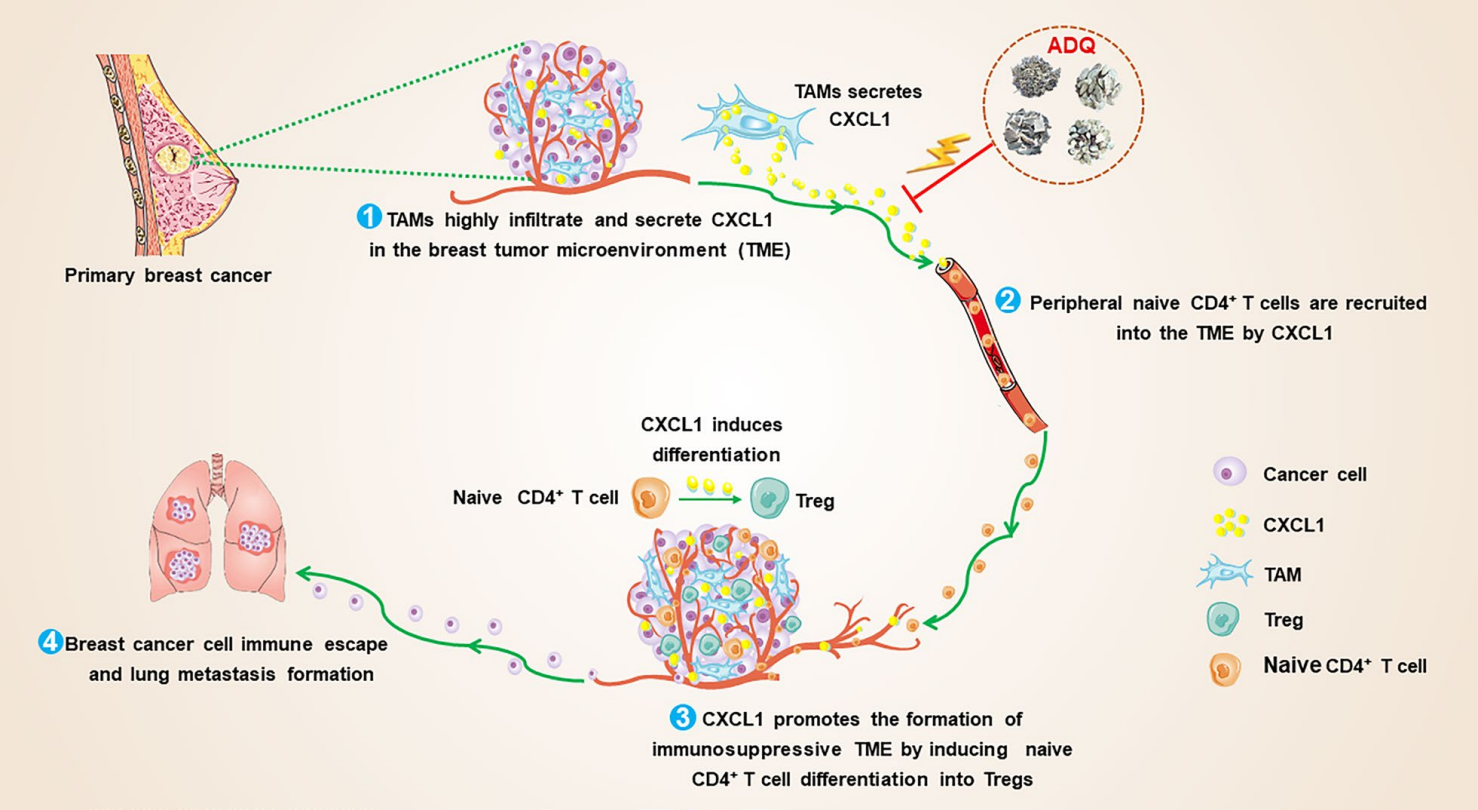
The biological mechanism of ADQ in inhibiting breast cancer immune escape and lung metastasis. TAM/CXCL1 signaling can promote the formation of the immunosuppressive TME by recruiting naive CD4^+^ T cells and inducing their differentiation into Tregs. ADQ suppresses breast cancer immune escape and lung metastasis by remodeling the immunosuppressive TME via inhibiting TAM/CXCL1-induced Treg differentiation and infiltration

In this study, we systematically demonstrated that CXCL1 derived from TAMs induced the establishment of the immunosuppressive TME by recruiting naive CD4^+^ T cells and promoting their differentiation into Tregs. ADQ suppressed the immune escape and lung metastasis of breast cancer by remodeling the immunosuppressive TME via blocking the TAM/CXCL1/Treg pathway (Fig. 7). This study not only provides preclinical evidence supporting the clinical use of ADQ in the treatment and prevention of breast cancer metastasis but also highlights TAM/CXCL1/Treg blockage as a promising treatment strategy for breast cancer immune escape and metastasis.

## Supplementary Information

**Additional file 1**. **Supplementary figures 1.** (The effect of ADQ on Th1 cell infiltration in the TME of mammary tumors), **2** (The effect of ADQ on the expression levels of iNOS and ARG1 in mammary tumor tissues was detected by Western blot assay), **3** (The effect of TAM-derived CXCL1 on the growth and metastasis of the co-injected breast cancer cells in the zebrafish breast cancer xenotransplantation model) and **Supplementary Table 1.** (ADQ exhibited no noticeable hepatotoxicity, nephrotoxicity, or hematotoxicity *in vivo*).

## Data Availability

The datasets used and/or analyzed during the current study are available from the corresponding author on reasonable request.

## Abbreviations

TCM: Traditional Chinese Medicine
ADQ: Aiduqing
TILs: Tumor-infiltrating lymphocytes
Tregs: Regulatory T cells
TME: Tumor microenvironment
TAMs: Tumor-associated macrophages
CTLs: Cytotoxic T lymphocytes
NKs: Natural killer cells
IL-6: Interleukin 6
ARG1: Arginase 1
ECM: Extracellular matrix
MMPs: Matrix metalloproteinases
RFS: Recurrence-free survival
OS: Overall survival
HSPCs: Hematopoietic stem and progenitor cells
MDSCs: Myeloid-derived suppressor cells
CSCs: Cancer stem cells
PMN: Pre-metastatic niche
ADCC: Antibody-dependent cell-mediated cytotoxicity
