# Correction to: Aiduqing formula inhibits breast cancer metastasis by suppressing TAM/CXCL1-induced Treg differentiation and infiltration

**DOI:** 10.1186/s12964-021-00802-2

**Published:** 2021-11-15

**Authors:** Jing Li, Shengqi Wang, Neng Wang, Yifeng Zheng, Bowen Yang, Xuan Wang, Juping Zhang, Bo Pan, Zhiyu Wang

**Affiliations:** 1 The Research Center of Integrative Cancer Medicine, Discipline of Integrated Chinese and Western Medicine, The Second Clinical College of Guangzhou University of Chinese Medicine, Guangzhou, Guangdong China; 2 Guangdong Provincial Key Laboratory of Clinical Research On Traditional Chinese Medicine Syndrome, Guangdong Provincial Academy of Chinese Medical Sciences, Guangdong Provincial Hospital of Chinese Medicine, Guangzhou, Guangdong China; 3 Guangdong-Hong Kong-Macau Joint Lab On Chinese Medicine and Immune Disease Research, Guangzhou University of Chinese Medicine, Guangzhou, Guangdong China; 4 The Research Center for Integrative Medicine, School of Basic Medical Sciences, Guangzhou University of Chinese Medicine, Guangzhou, Guangdong China; 5 State Key Laboratory of Dampness, Syndrome of Chinese Medicine, The Second Affiliated Hospital of Guangzhou University of Chinese Medicine, Guangzhou, 510120 China

## Correction to: Cell Commun Signal19, 89 (2021) 10.1186/s12964-021-00775-2

Following publication of the original article [1], the authors found an error in Fig. 6D. Unintentionally, the same lung was mistakenly displayed twice in the TAM group (the upper and the lower image). In order to further verify the accuracy of the study, we would like to provide the revised Fig. 6D. This correction does not influence the description, interpretation or the original conclusions of the article.

**Fig. 6:**
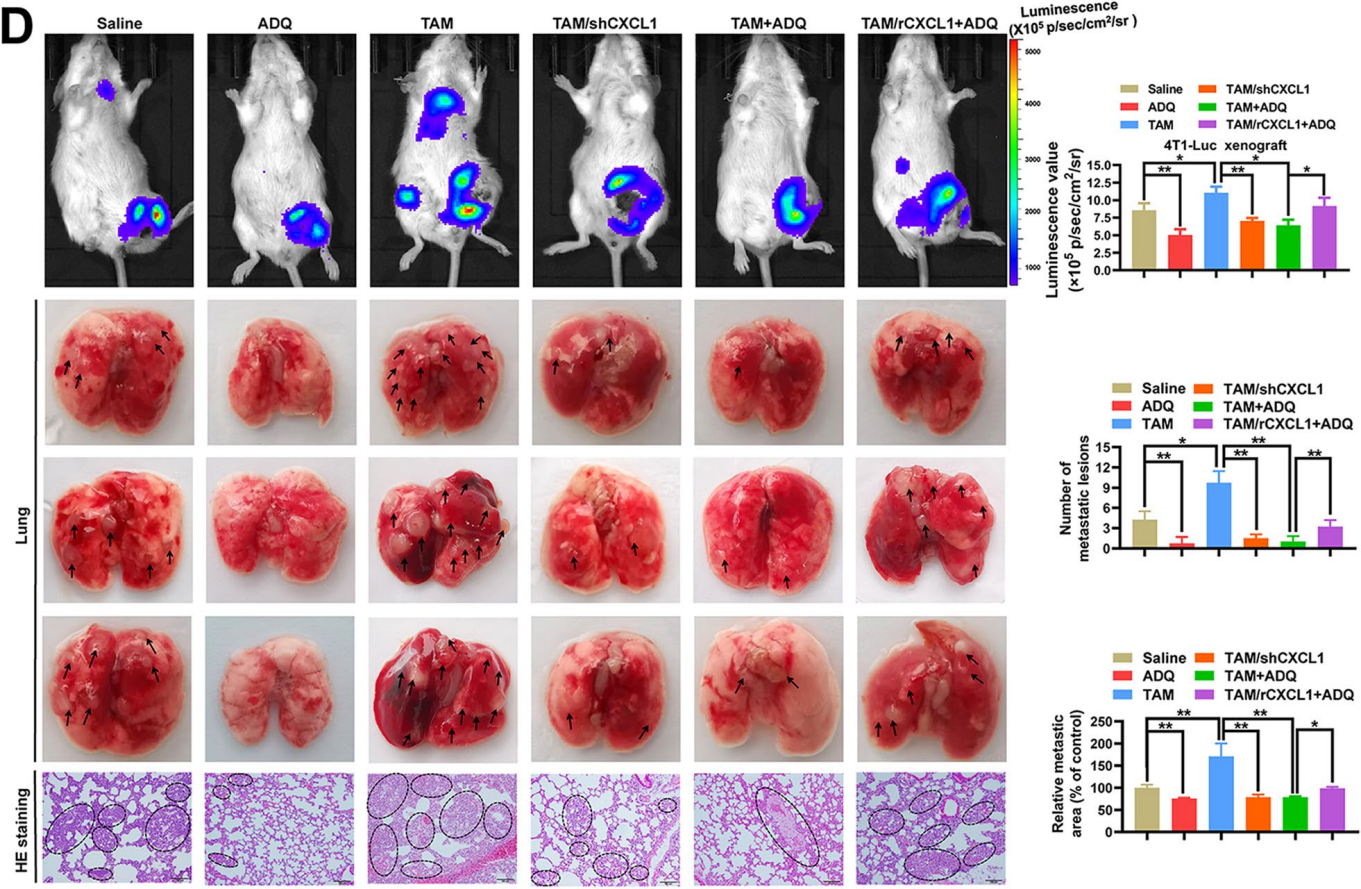
**D** Both the in vivo imaging assay and lung HE staining assay suggested that ADQ dramatically suppressed the lung metastasis of mammary tumors by inhibiting the TAM/CXCL1 pathway. Scale bar = 100 μm. N = 3. Arrows and circles indicate the metastatic tumor foci in murine lungs

## References

[1] Li J, Wang S, Wang N (2021). Aiduqing formula inhibits breast cancer metastasis by suppressing TAM/CXCL1-induced Treg differentiation and infiltration. Cell Commun Signal.

