# Supplementary material for: Aiduqing formula inhibits breast cancer metastasis by suppressing TAM/CXCL1-induced Treg differentiation and infiltration
Source: Cell Commun Signal. 2021 Aug 30;19:89. doi: 10.1186/s12964-021-00775-2 (PMC8404313; doi:10.1186/s12964-021-00775-2)
Supplement: Supplementary file 2 — Additional file 1. Supplementary figures 1. (The effect of ADQ on Th1 cell infiltration in the TME of mammary tumors), 2 (The effect of ADQ on the expression levels of iNOS and ARG1 in mammary tumor tissues was detected by Western blot assay), 3 (The effect of TAM-derived CXCL1 on the growth and metastasis of the co-injected breast cancer cells in the zebrafish breast cancer xenotransplantation model) and Supplementary Table 1. (ADQ exhibited no noticeable hepatotoxicity, nephrotoxicity, or hematotoxicity in vivo). [file 12964_2021_775_MOESM1_ESM.docx]

**Aiduqing formula inhibits breast cancer** **metastasis by suppressing TAM/CXCL1-induced Treg differentiation and infiltration**

**Supplementary Figures**

**
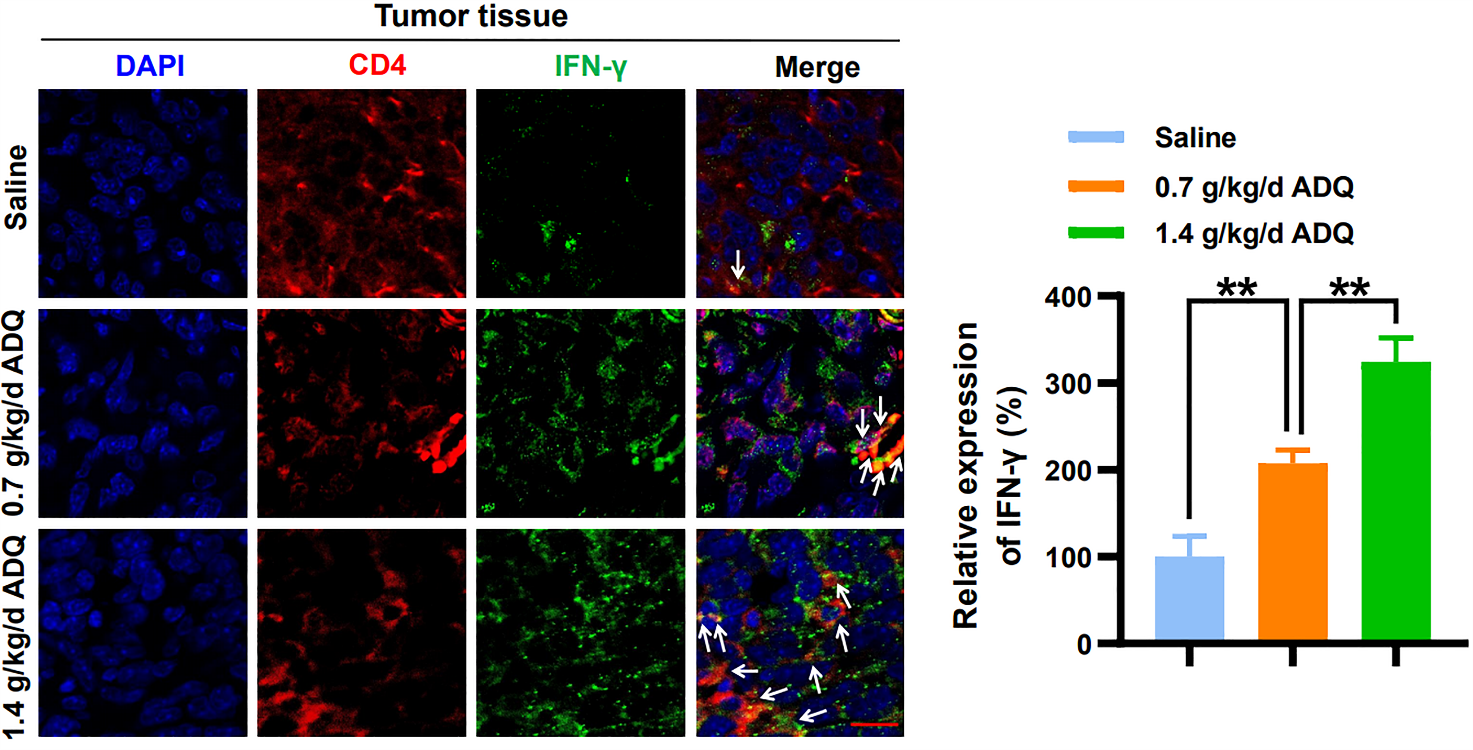
**

**Supplementary Figure 1. The effect of ADQ on Th1 cell infiltration in the TME of mammary tumors.** CD4 and IFN-γ expressions in mammary tumor tissues were detected by the immunofluorescence assay. Arrows indicate Th1 cells within the TME of mammary tumors. Scale bar = 10 μm. N = 3. ^**^*p* < 0.01.

**
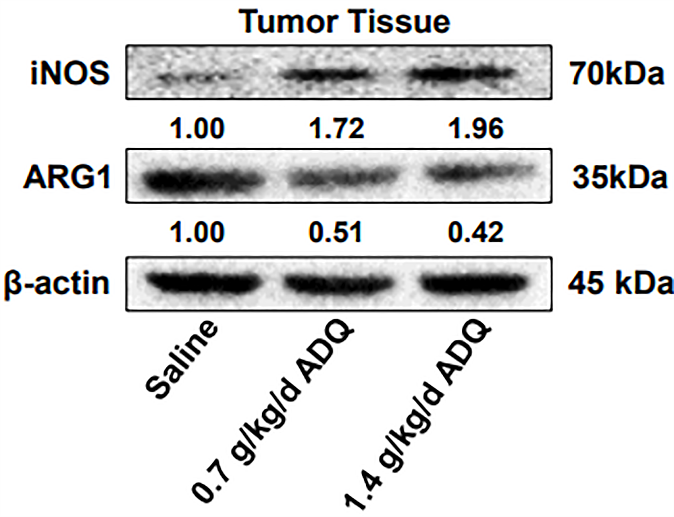
**

**Supplementary Figure 2. The effect of ADQ on the expression levels of iNOS and ARG1 in mammary tumor tissues was detected by Western blot assay.**

**
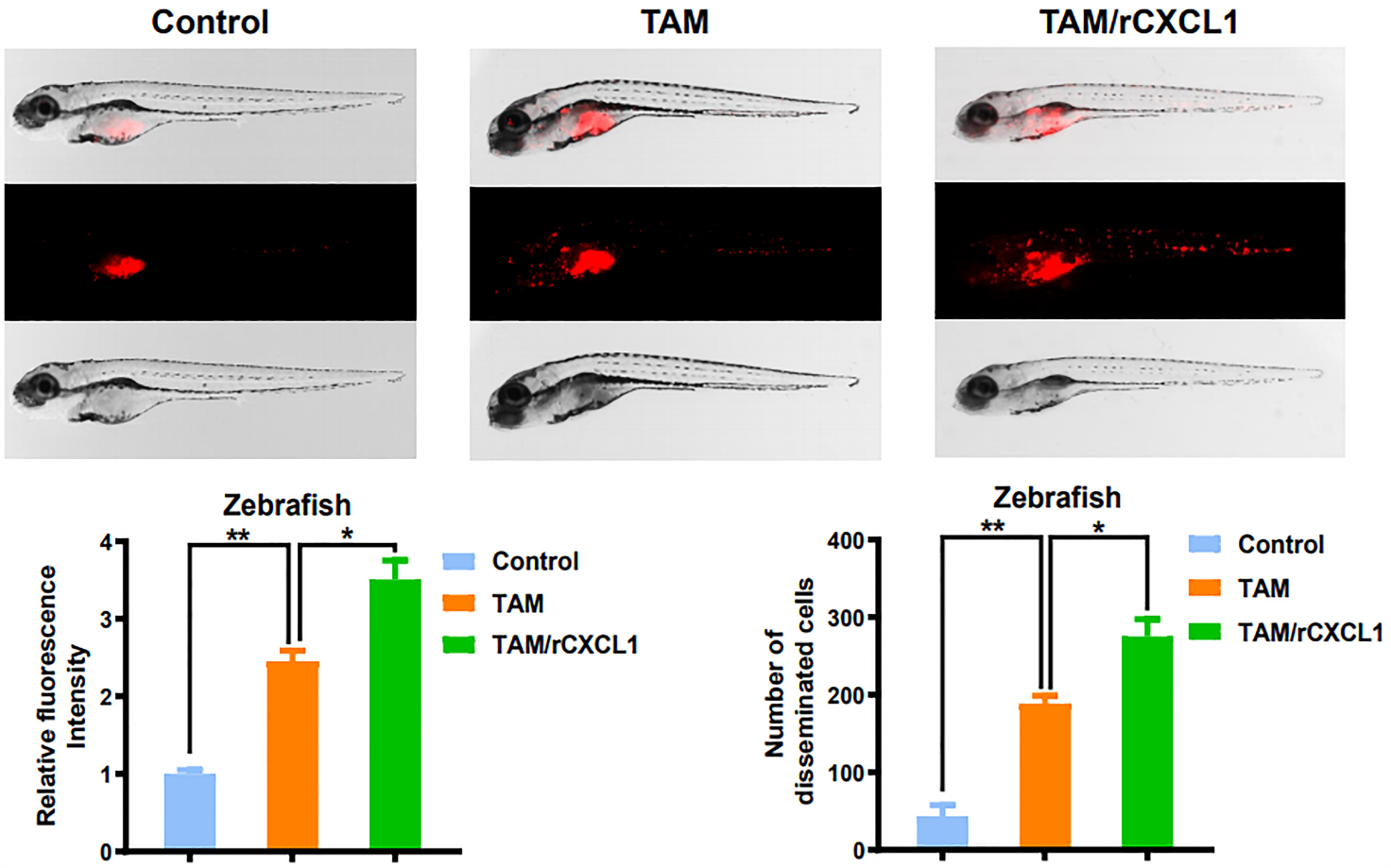
**

**Supplementary Figure 3. The effect of TAM-derived CXCL1 on the growth and metastasis of the co-injected breast cancer cells in the zebrafish breast cancer xenotransplantation model.** A detailed description of the zebrafish breast cancer xenotransplantation assay is provided in the Supplementary Materials and Methods. N = 3. ^*^*p* < 0.05. ^**^*p* < 0.01.

**Supplementary Table**

**Supplementary Table 1. ADQ exhibited no noticeable hepatotoxicity, nephrotoxicity, or hematotoxicity *in vivo*.**

| **Blood biochemical values** | **Saline** | **0.7 g/kg/d ADQ** | **1.4 g/kg/d ADQ** | ***p* values** |
| --- | --- | --- | --- | --- |
| **ALT(U/L)** | **21.7 ± 0.6** | **22.3 ± 2.3** | **23.3 ±1.5** | **0.495** |
| **AST(U/L)** | **163.3 ± 3.8** | **163.3 ± 2.5** | **170.0 ± 9.5** | **0.366** |
| **Urea(mmol/L)** | **3.3 ± 0.2** | **3.3 ± 0.1** | **3.4 ± 0.3** | **0.740** |
| **Cr(µmol/L)** | **19.3 ± 0.6** | **20.0 ± 1.0** | **20.3 ± 1.5** | **0.562** |
| **UA(µmolL)** | **191.7 ± 0.6** | **185.7 ± 10.1** | **201.0 ± 13.0** | **0.219** |
| **WBC(10^9^/L)** | **2.4 ± 0.3** | **2.4 ± 0.2** | **2.5 ± 0.3** | **0.669** |
| **RBC(10^12^/L)** | **9.9 ± 0.4** | **9.5 ± 0.5** | **9.9 ± 0.5** | **0.620** |
| **Hb(g/L)** | **151.7 ± 10.1** | **146.0 ± 9.5** | **152.0 ± 10.4** | **0.726** |

**ALT, Alanine transaminase; AST, Aspartate aminotransferase; Cr, Creatinine; UA, Uric acid; WBC, White blood cell; RBC, Red blood cell; Hb, Hemoglobin.**

**Supplementary Materials and Methods**

**Zebrafish breast cancer xenotransplantation assay**

The zebrafish xenotransplantation model was applied to investigate the effect of TAM-derived CXCL1 on the growth and metastasis of breast cancer *in vivo*. The AB strain zebrafish was obtained from China Zebrafish Resource Center (Wuhan, China). Zebrafish maintenance and egg production were carried out as we previously described [[1](#_ENREF_3_1)]. To establish the 4T1-Dil zebrafish xenotransplantation model, 4T1 cells were collected in DMEM medium and labeled red fluorescence by 5 μM 1, 1′-Dioctadecyl-3, 3, 3′, 3′-tetra-methylindocarbocyanine perchlorate (DiI, Sigma-Aldrich). Juvenile zebrafish (48 h post fertilization) were randomized into 3 groups, including the control group, TAM group, and TAM/rCXCL1 group. For the control group, 300 4T1-Dil cells were suspended in 50 nl DMEM complete medium and injected into the subintestinal vessels of each zebrafish by a microinjector. For the TAM group and TAM/rCXCL1 group, 300 4T1-Dil cells were co-injected with 900 Raw264.7-derived TAMs or 900 Raw264.7/rCXCL1-derived TAMs, respectively. Juvenile zebrafishes bearing breast cancer cells were further incubated in 6-well plates for 72 h. Then, the Dil-stained 4T1 cells in zebrafish were observed under a fluorescence microscope (Nikon Eclipse C1, Tokyo, Japan).

**References**

1. Jiao L, Wang SQ, Zheng YF, Wang N, Yang BW, Wang DM, et al. Betulinic acid suppresses breast cancer aerobic glycolysis via caveolin-1/NF-kappa B/c-Myc pathway. Biochem Pharmacol. 2019;161:149-62.
